# Management Strategies in Septic Coagulopathy: A Review of the Current Literature

**DOI:** 10.3390/healthcare11020227

**Published:** 2023-01-12

**Authors:** Piotr F. Czempik, Agnieszka Wiórek

**Affiliations:** 1 Department of Anaesthesiology and Intensive Care, Faculty of Medical Sciences in Katowice, Medical University of Silesia, 40-752 Katowice, Poland; 2 Transfusion Committee, University Clinical Center, Medical University of Silesia, 40-752 Katowice, Poland

**Keywords:** antithrombin, coagulopathy, disseminated intravascular coagulation, heparin, management, sepsis, thrombomodulin, treatment

## Abstract

One of the ‘organs’ that can be affected by sepsis is the coagulation system. Coagulopathy in sepsis may take the form of sepsis-induced coagulopathy (SIC) or sepsis-associated disseminated intravascular coagulation (DIC). It is important to identify SIC early, as at this stage of coagulopathy anticoagulants may be of the greatest benefit. The most recent diagnostic scoring systems for septic coagulopathy come from the International Society on Thrombosis and Hemostasis and the Japanese Association for Acute Medicine. Recommendations regarding the management of septic coagulopathy differ between organizations. Moreover, septic coagulopathy is an area of intense research in recent years. Therefore we searched three databases to review the most recent management strategies in septic coagulopathy. The mainstream management strategies in septic coagulopathy include the causal treatment of sepsis, unfractionated heparin, low-molecular-weight heparin, antithrombin, and recombinant human thrombomodulin. The last two have been associated with the highest survival benefit. Nevertheless, the indiscriminate use of these anticoagulants should be avoided due to the lack of mortality benefit and increased risk of bleeding. The early diagnosis of SIC and monitoring of coagulation status during sepsis is crucial for the timely management and selection of the most suitable treatment at a time. New directions in septic coagulopathy include new diagnostic biomarkers, dynamic diagnostic models, genetic markers for SIC management, and new therapeutic agents. These new research avenues may potentially result in timelier SIC diagnosis and improved management of all stages of septic coagulopathy by making it more effective, safe, and personalized.

## 1. Introduction

Sepsis is defined as ‘a life-threatening organ dysfunction caused by a dysregulated host response to infection’ [1]. Septic shock is defined as a subtype of sepsis, with hemodynamic and metabolic abnormalities, and is associated with increased mortality [1]. One of the ‘organs’ that can be affected by sepsis is the coagulation system.

Coagulopathy in sepsis may take the form of sepsis-induced coagulopathy (SIC) (early stage) [2] or sepsis-associated disseminated intravascular coagulation (DIC) (late stage). Disseminated intravascular coagulation is defined by the International Society on Thrombosis and Hemostasis (ISTH) as ‘an acquired syndrome characterized by the intravascular activation of coagulation with loss of localization arising from different causes. It can originate from and cause damage to the microvasculature, which, if sufficiently severe, can produce organ dysfunction’ [3]. These two clinical syndromes differ in pathophysiology. A major role in the pathophysiology of SIC is attributed to decreased fibrinolysis, caused by the overproduction of plasminogen activator inhibitor-1 (PAI-1), leading to hypercoagulation [4]. It is important to identify SIC early because anticoagulants at this stage may be of the greatest benefit. The transition from SIC to overt sepsis-associated DIC is associated with the massive consumption of coagulation factors and platelets; hence, bleeding complications occur, and anticoagulants at this stage may be of no benefit [5]. Recommendations regarding the management of SIC/sepsis-associated DIC differ between organizations [6,7]. There are several management strategies that can be employed in patients with SIC and sepsis-associated DIC. This topic is an area of active research, with a great number of new publications in the last 5 years.

Therefore, we searched three databases (PubMed, Embase, Cochrane Library) to review the most recent management strategies in SIC and sepsis-associated DIC. We reviewed publications no more than 5 years old (e.g., 2018–present). If there was no current literature on the topic, we used the most recent publications. As far as the management of septic coagulopathy is concerned, it is very important to be able to identify this syndrome early; therefore, we present diagnostic scoring systems in the first section of this review paper.

## 2. Diagnostic Criteria for SIC and Sepsis-Associated DIC

The accurate diagnosis of SIC and sepsis-associated DIC allows physicians to determine which patients may benefit from therapy. Specific therapeutic approaches aimed at coagulopathy are of no benefit to sepsis patients without coagulation abnormalities; moreover, they may have harmful effects, as shown for activated protein C (APC), what resulted in its withdrawal from the market [8].

### 2.1. International Society on Thrombosis and Hemostasis (ISTH)

The ISTH published SIC diagnostic criteria in 2017. These criteria include platelet count (PLTs), the international normalized ratio (INR), and the Sepsis Organ Failure Assessment (SOFA) score (Table 1) [2]. This scoring system has been specifically designed for sepsis. According to the international consensus, sepsis is defined as ‘a life-threatening organ dysfunction caused by a dysregulated host response to infection’ [1]. The Sepsis Organ Failure Assessment (SOFA) scale measures organ dysfunction caused by sepsis. Septic shock is currently defined as ‘a subset of sepsis in which underlying circulatory and cellular metabolism abnormalities are profound enough to substantially increase mortality’. Cardiovascular insufficiency is one of the factors measured by the SOFA score.

Sepsis-induced coagulopathy is diagnosed when the total SIC score is ≥4. Since their establishment, the newly proposed SIC criteria have been validated [9]. Although, as mentioned earlier, the most important role in the pathophysiology of SIC is attributed to decreased fibrinolysis, with a resultant hypercoagulable state, the ISTH SIC classification system does not include any parameter that measures the fibrinolytic system activity. For the measurement of fibrinolytic system activity, viscoelastic tests have to be used; however, these methods are not widely available. Viscoelastic tests will be discussed later in this section.

In its most recent publication on the topic, the ISTH proposes a two-step diagnostic approach to septic coagulopathy [10]. In the first step, sepsis patients with thrombocytopenia are screened with the SIC scale. The second step is to screen patients with positive SIC scores (≥4) with the overt DIC score. The rationale for this two-step approach is that SIC clinically precedes sepsis-associated DIC. It was shown that 93.9% of patients who were diagnosed with SIC went on to develop sepsis-associated DIC within the next 2–4 days [11]. Hence, SIC and overt DIC represent a continuum. The onset of SIC is the moment when anticoagulant therapy is of the greatest benefit [10]. The overt DIC in this algorithm is diagnosed based on the ISTH criteria from 2001 (Table 2) [3].

**Figure 1:**
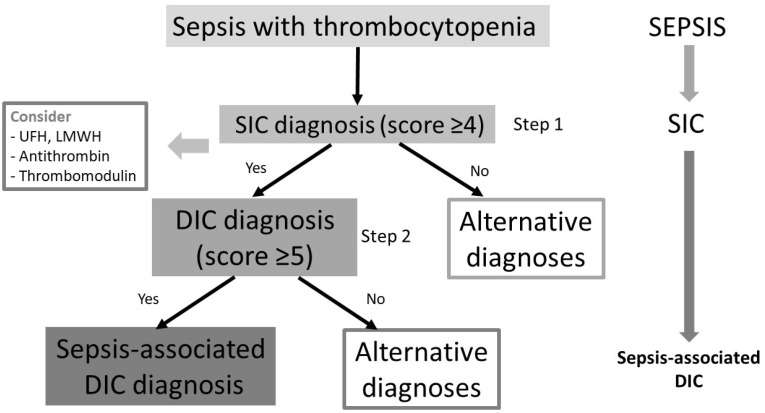
Two-step diagnostic approach to septic coagulopathy: DIC—disseminated intravascular coagulation; SIC—sepsis-induced coagulopathy; LMWH—low-molecular-weight heparin; UFH—unfractionated heparin. Based on [10].

The ISTH DIC diagnostic scoring system from 2001 has not been specifically designed for sepsis. This scoring system is used in patients with various pathologies that are associated with the development of DIC: sepsis (bacteria, viruses, and parasites), malignancy (solid tumors and hematological malignancies), trauma, pancreatitis, obstetric complications (placental abruption, placenta previa, amniotic fluid embolism, intrauterine death, eclampsia, the HELLP (hemolysis, elevated liver enzymes, and low platelets) syndrome), severe liver failure, toxic and immunological insults (e.g., snake bites, recreational drug use, ABO transfusion incompatibility, and transplant rejection) [12,13]. For fibrin-related markers (e.g., D-dimers and fibrin degradation products), local laboratory-specific cut-off values should be used. A score of <5 is suggestive of non-over DIC, whereas a score of ≥5 is compatible with overt DIC. The two-step diagnostic approach to septic coagulopathy is presented in Figure 1.

### 2.2. Japanese Association for Acute Medicine (JAAM)

These diagnostic criteria are specifically designed for sepsis- and trauma-associated DIC [5]. In this classification system, the criterion for fibrinogen was removed, whereas the criterion for systemic inflammatory response syndrome (SIRS) was added, as opposed to the older ISTH overt DIC definition, which included fibrinogen concentration while still leaving the criterion for fibrin degradation products [14]. The scoring system is presented in Table 3.

The criteria for the systemic inflammatory response syndrome used in this classification system include the temperature (<36 °C or >38 °C), the heart rate (>90 beats/min.), the respiratory rate (>20 breaths/min. or PaCO_2_ < 35 mmHg/4.3 kPa), and white cell count (>12 × 10^9^/L or <4 × 10^9^/L or 10% immature forms present). A score of ≥4 supports the diagnosis of DIC.

In a recent study, Tanaka et al. aimed to compare the three most recognized diagnostic scoring systems for septic coagulopathy: the ISTH overt DIC score, the JAAM DIC score, and the ISTH SIC score [15]. The authors divided septic population into two subgroups of patients who required and did not require vasopressors and screened them towards the fulfillment of the SIC criteria. The authors concluded that the ISTH SIC score may be the simplest method among these instruments, and additionally, it is based on the SOFA score, not SIRS criteria, which have been challenged by the most recent sepsis guidelines [6,16].

### 2.3. Thromboelastometry and Thromboelastography in SIC and Sepsis-Induced DIC

An excessive inflammatory response in sepsis patients activates coagulation and leads to an early hypercoagulable state, followed by a secondary consumption of coagulation factors and platelets, resulting in a severe hypocoagulable state [17]. Boscolo et al. analyzed studies focused on sepsis patients hospitalized in the ICU or emergency department and aimed to analyze if viscoelastic testing can be used to stratify mortality based on coagulation profiles and diseases, as this was an issue not directly resolved in the previous studies [18]. The authors observed that there were major alterations in both rotational thromboelastometry (ROTEM) and thromboelastography (TEG) parameters. Prolonged INTEM clotting time (CT) and TEG R-time, INTEM clot formation time (CFT) and TEG K-time, EXTEM CT, and reduced EXTEM maximum clot formation (MCF) were associated with an increased mortality risk (*p* < 0.05 for all). Additionally, the frequency of hypocoagulable state on admission was significantly higher in non-survivors [18]. In another retrospective study, Lou et al. aimed to assess the diagnostic value of point-of-care viscoelastic testing for SIC, focusing on TEG parameters [19]. Their comparison of SIC and non-SIC sepsis patients revealed that the R-time and K-time values were longer in the SIC group (*p* = 0.001), whereas the angle and maximum amplitude (MA) were significantly lower in the SIC group (*p* < 0.0001) [19]. For prediction of SIC, the ROC analysis of TEG parameters revealed the AUC value of 0.91 for the K-time (95% CI 0.82–0.99). For the exclusion of SIC, AUC values for the angle and MA were 0.89 (95% CI 0.81–0.98) and 0.88 (95% CI 0.79–0.97), respectively (*p* < 0.05 for all). The R-time proved to be not useful in SIC diagnostics [19].

## 3. Management Strategies in SIC and Sepsis-Associated DIC

### 3.1. Causal Treatment of Sepsis

The British guidelines for the diagnosis and management of DIC state that ‘the cornerstone of the treatment of DIC is treatment of the underlying condition’ [12]. Early antibiotic therapy is the key therapy in sepsis patients. The Surviving Sepsis Campaign (SSC) guidelines recommend the administration of an antimicrobial agent within an hour of diagnosis of sepsis or septic shock. Empiric broad-spectrum antimicrobial therapy may differ depending on the most common microbes and their resistance patterns in the local setting—the so-called microbiological map [6]. The newest edition of the SSC guidelines published in 2021 upgraded the importance of infection source control. If the source of infection is amendable to removal, it should be removed as soon as it is logistically possible. If the probable source of infection is a vascular line, alternative vascular access should be secured, and the vascular line that is the probable source of infection should be removed immediately [6].

Another key therapy in the management of sepsis patients is fluid resuscitation. The most recent edition of the SSC guidelines downgraded the importance of the initial fluid bolus of 30 mL/kg of balanced crystalloid, paying more attention to fluid responsiveness measured with dynamic methods [6].

### 3.2. Unfractionated Heparin (UFH)

It is important to mention that UFH in sepsis patients may have much more than only anticoagulant effects. Heparin has various immunomodulatory properties: It inhibits lung inflammation via the inactivation of NF-κB [20], in addition to inhibiting neutrophil recruitment [21] and LPS-induced inflammatory mediators [22], and it binds to histones [23]. Heparin may also protect glycocalyx from shedding [24,25]. The most recent study analyzing the effect of UFH in patients with SIC was performed in 2014 [26]. In this small (n = 37), prospective clinical study, the dose of UFH was 70 u/kg/24 h as a continuous infusion, and the dose was adjusted to aim for 2–3-fold prolongation of activated partial thromboplastin time (aPTT). The intervention drug decreased the hypercoagulable state, as judged by the concentration of prothrombin fragments 1 and 2 (F1 + 2) and thrombin-antithrombin complexes (TATs), the time of mechanical ventilation, and hospitalization in the ICU. The retrospective analysis of a large database of SIC (ISTH) patients performed in 2022 showed that UFH given subcutaneously or via continuous intravenous infusion, in prophylactic or therapeutic doses, reduced 28-day mortality (hazard ratio (HR) 0.32, 95% CI 0.26–0.41, *p* < 0.001) and hospital mortality (HR 0.38, 95% CI 0.31–0.47, *p* < 0.001), with a favorable safety profile in the context of intracranial and gastrointestinal hemorrhage [27]. Another retrospective analysis of a large database showed that early administration of UFH in prophylactic doses (at least five doses) was associated with decreased in-hospital mortality (HR 0.70, 95% CI 0.56–0.87, *p* < 0.001) [28].

### 3.3. Low-Molecular-Weight Heparin (LMWH)

Some of the newest reports regarding the potential superiority of LMWH over UFH for prophylaxis and treatment of SIC come from the experience gained during the novel coronavirus disease (COVID-19) pandemic. A retrospective analysis of COVID-19 patients showed that the 28-day mortality of patients with SIC scores (ISTH) of ≥4, who received LMWH, was lower in patients who did not receive LMWH (40.0 vs. 64.2%, *p* = 0.029) [29]. Moreover, the recently concluded HEP-COVID randomized clinical trial enrolled COVID-19 patients with increased D-dimer concentration or SIC scores of ≥4 to examine the difference between therapeutic dose, intermediate dose, and standard prophylactic LMWH dose subgroups [30]. The primary outcome was venous or arterial thromboembolism or death from any cause. The principal safety outcome was major bleeding at 30 days. In the subgroup of patients treated with the therapeutic-dose LMWH, a reduction in thromboembolism was noted (0.37 (95% CI 0.21–0.66, *p* < 0.0001)). Overall, the results showed that the therapeutic dose compared with the prophylactic dose reduced the occurrence of thromboembolism and death without increasing major bleeding among COVID-19 inpatients with increased SIC scores; however, these observations were only shown in the non-ICU population [30].

### 3.4. Antithrombin (AT)

Antithrombin is a potent physiological anticoagulant. Moreover, AT binds to heparan sulfate present in the glycocalyx layer [31,32]. The most recent Japanese Clinical Practice Guidelines for Management of Sepsis and Septic Shock suggest the use of AT in patients with sepsis-associated DIC whose AT activity is ≤70% [33]. This suggestion was based on four studies, one of which showed reduced mortality [34], while the other three showed no effect on mortality with improvement in DIC scores [35,36,37]. The problem with studies on the pharmacological treatment of sepsis-associated DIC is that not all studies accurately identified DIC patients; hence, the beneficial effect might not be shown [38,39]. In 2016, the inverse probability of the treatment-weighted propensity score analysis on 1784 patients diagnosed with DIC showed a statistically significant association between AT supplementation and lower in-hospital all-cause mortality (OR 0.75, 95%CI 0.57–0.98, *p* = 0.034) [40]. In 2018, the results of a network meta-analysis in patients with sepsis-associated DIC were published. The authors compared four different anticoagulants and a placebo. The results showed that AT compared with the placebo was associated with a five-fold higher likelihood of sepsis-associated DIC resolution (OR 0.20, 95% credible intervals 0.05–0.81) [41]. In 2019, Tanaka et al. published the results from a nationwide registry study on the use of AT in sepsis-associated DIC. Almost 3000 patients in the registry had mostly abdominal origin of sepsis, and most of them required vasopressors. Although the analysis of all the included patients did not show any effect of AT on in-hospital mortality, the subanalysis of patients diagnosed with sepsis-associated DIC (ISTH criteria) demonstrated an independent association between AT and/or recombinant human thrombomodulin (rhTM) and lower in-hospital mortality than other anticoagulants (HR 0.74 (95% CI 0.60–0.92) [42]. A post hoc subgroup analysis of these registry data showed survival benefits for the use of AT without concomitant heparin in sepsis patients with higher predicted mortality and most severe coagulopathy [43]. As far as the optimal threshold for starting the AT therapy is concerned, in-hospital mortality was significantly reduced only in patients with very low antithrombin activity ≤43% (adjusted HR 0.60, 95%CI 0.37–0.99; *p* = 0.045) [44]. The therapeutic goal for AT supplementation was activity ≥70%, optimally 80% [45].

### 3.5. Activated Protein C (APC)

Performed in 2011, the PROWESS SHOCK study aimed to evaluate the initial promising results of its predecessor, the PROWESS trial. It was supposed to assess the clinical effectiveness and safety of APC, the only novel anti-sepsis agent to successfully complete the phase 3 trial [46]. However, this follow-up study showed no effect of APC in reducing the risk of death in sepsis patients. Moreover, the use of APC was associated with a higher risk of bleeding, with RR 1.45 (95% CI 1.08–1.94, *p* < 0.05), and no effect on the risk of any other serious adverse event (RR 1.04, 95% CI 0.92–1.18, *p* < 0.05) [46]. This evidence resulted in taking the potentially immunomodulating agent off the market [47].

### 3.6. Thrombomodulin (TM)

Thrombomodulin is an endothelial anticoagulant cofactor promoting the thrombin-mediated activation of protein C [48]. In the SCARLET trial, investigators defined septic-associated coagulopathy (SAC) as INR > 1.4, with no other explanation for this abnormality than sepsis, thrombocytopenia in the range 30–150 × 10^9^/L or >30% platelet decrease in 24 h, and respiratory or hemodynamic dysfunction. In this randomized, controlled trial (RCT), patients were randomized to receive a bolus of rhTM or a placebo for 6 days. The 28-day all-cause mortality did not differ significantly between the intervention and the placebo groups (26.8% vs. 29.4%, *p* = 0.32). However, the post hoc analysis of the SCARLET trial suggests that patients who had features of SAC at the time of administration of the first dose of rhTM and did not receive heparin may benefit from it [49]. Another post hoc subgroup analysis of the same trial showed that absolute risk reduction was greater in patients with higher baseline thrombin generation biomarker concentration (F1 + 2 or TAT) [50]. An analysis of three multicenter observational studies showed that rhTM was associated with a lower rate of 28-day mortality (adjusted risk difference RD—17.8% (95% CI—28.7 to—6.9%)) and in-hospital mortality (adjusted RD—17.7% (95% CI—27.6 to—7.8%)), but only in sepsis patients with a high concentration of D-dimers (Me 51900 (IQR 35200–113000) ng/mL) and fibrin degradation products (Me 120200 (IQR 79200–266000) ng/mL). The study was interesting because four coagulation phenotypes of sepsis were identified based on the laboratory parameters of hemostasis: severe sepsis and severe coagulopathy, severe sepsis and moderate coagulopathy, moderate sepsis with coagulopathy, and mild sepsis without coagulopathy [51]. A post hoc analysis of patients with sepsis-associated DIC registered in a nationwide multicenter Japanese database showed that the combined therapy with AT and rhTM does not present additional benefits in terms of survival [52]. Another post hoc analysis from a post-marketing surveillance database from Japan showed that in patients with severe thrombocytopenia (<50 × 10^9^/L) and AT deficiency (activity < 50%), the concomitant use of rhTM and AT reduced 28-day mortality (HR 0.62, 95%CI 0.39–0.98) [53].

### 3.7. Tissue Factor Pathway Inhibitor (TFPI)

A tissue factor pathway inhibitor is a plasma protease inhibitor that blocks the initiation phase of thrombin generation induced by the tissue factor (TF) [54]. The in vivo administration of recombinant TFPI (rTFPI) in experimental animal models prevented thrombosis and fibrin deposition on the subendothelial matrix, reduced mortality from Escherichia coli-induced sepsis, and protected against DIC development [13]. The elevated levels of TFPI have been found in patients with sepsis-associated DIC in conjunction with elevated TF levels, suggesting a relative deficiency of TFPI to neutralize the TF pathway activation. Since coagulation activation in sepsis-associated DIC is primarily mediated through the TF/FVIIa pathway and the overexpression of TF compared with TFPI, the substitution of TFPI seems a rational treatment approach [13].

There is a paucity of new data regarding the use of TFPI in sepsis management, as most of the clinical trials come from the years 2000–2006 [55,56,57]. There are foundations for further investigation based on the role of endothelial cells in maintaining intravascular patency through their anticoagulant properties. Endothelial cells synthesize proteoglycans, a component of the glycocalyx, which bind and potentiate plasma anticoagulant proteins, including TFPI and AT [58]. Under inflammatory and septic conditions, endothelial cells lose their anticoagulant properties. Injury to endothelial cells and the destruction of the glycocalyx due to the suboptimal synthesis of TFPI may induce the activation of coagulation, accelerating the development of sepsis-associated DIC [59]. The study by Walborn et al. aimed to quantify endothelial function, including endogenous anticoagulants such as TFPI and protein C, in the plasma of patients with sepsis and DIC [60]. The researchers wanted to determine how these factors relate to the severity of illness and the outcome. In their study group, TFPI concentration was increased in patients with overt DIC (mean 110, SD ± 90), non-overt DIC (mean 104, SD ± 69) vs. no DIC (mean 95, SD ± 58), and healthy controls (mean 61, SD ± 19) ng/mL (*p* < 0.05). Further analysis showed higher TFPI concentration in the non-survivors; this result, however, was not statistically significant. The authors concluded that although TFPI did not show significant variation based on mortality, the increased concentration of TFPI in patients with sepsis and DIC, compared with healthy controls, may emphasize the role of endogenous anticoagulants in the disease process. The measurement of functional TFPI levels, in addition to protein levels, may provide further insight into the role of TFPI in sepsis-associated DIC [60].

### 3.8. Transfusion of Blood Components

The British guidelines for the diagnosis and management of DIC suggest that in patients with DIC, the transfusion of PLTs (trigger < 50 × 10^9^/L) and/or fresh-frozen plasma (FFP) should be used if bleeding occurs or invasive procedures are planned. Moreover, if there are concerns with fluid overload, coagulation factor concentrates can be used (e.g., prothrombin complex concentrate, and fibrinogen concentrate). However, clinicians should bear in mind that DIC leads to the consumption of all coagulation factors [12]. A recent, prospective, open-label RCT in children with severe sepsis or septic shock showed that a combination of fresh-frozen plasma, low-dose heparin, and tranexamic acid was associated with better survival and prevented progression to overt sepsis-associated DIC, with no increase in bleeding [61].

### 3.9. Therapeutic Plasma Exchange (TPE)

In a prospective, randomized study, it was shown that TPE may outperform UFH in the treatment of patients with sepsis-associated DIC based on laboratory (e.g., markers of endothelial injury) and clinical (e.g., 28-day cumulative survival, acute kidney injury, acute respiratory distress syndrome, and bleeding events) outcomes. The possible mechanism is suggested to be improved endothelial function [62].

## 4. Future Directions in Management of SIC and Sepsis-Associated DIC

### 4.1. Dynamic/Continuous SIC/Sepsis-Associated DIC Prediction Scores

In the current era of dynamic development of artificial intelligence and global computerization, there is an ongoing effort to develop and validate machine-learning models for the early dynamic prediction of SIC in ICU patients. These dynamic models could help clinicians recognize the early warning signs of SIC and sepsis-associated DIC in patients from the moment of ICU admission. Zhao et al. used two sizeable critical care databases, the Medical Information Mart for Intensive Care (MIMIC IV) and the eICU Collaborative Research Database (eICU-CRD), to compile two models that were able to predict SIC better than conventional logistic regression and known SIC scores. Moreover, by computing the medical data of almost 50 000 patients, the authors developed a continuous prediction score based on 88 variables analyzed on each day when patients were septic. The authors intend to make this continuous prediction score into a web-based tool that could be accessed by physicians worldwide [63].

### 4.2. New Markers for SIC

The early activation of coagulation or the inhibition of fibrinolysis in sepsis cannot be detected by using the standard laboratory tests of coagulation. Therefore, new markers for SIC are needed. Four new markers were researched in a pediatric population: soluble TM, TAT, the α2-plasmin inhibitor–plasmin complex (PIC), and tissue plasminogen activator–inhibitor complex (t-PAIC). The authors showed that only t-PAIC was significantly different between the subgroups with and without SIC (*p* = 0.001) [64]. In 2022, Ishikura et al. published the results of an observational study on the C2PAC index, a ratio of soluble type C lectin-like receptor 2 (sCLEC-2) concentration and PLTs, for the prediction of sepsis-associated DIC [65]. The CLEC-2 was present on the surface of activated PLTs. Standard test tube anticoagulants, such as EDTA or sodium citrate, had no impact on the sCLEC-2 concentration. The authors emphasized that in patients with sepsis, PLT activation may occur before thrombocytopenia ensues and may signal the pre-DIC phase. The authors suggested that a sustained rise in the C2PAC index seems to be a good predictor of progression to the sepsis-associated DIC (AUROC 0.953) [65].

### 4.3. Toward Personalized Diagnostics

The development of individualized, “tailored”, goal-directed medicine has not omitted the topic of sepsis outcome prognostication. Genetic markers may play a role in SIC management in the future. MicroRNAs have been widely studied as biomarkers for several disorders, but little is known regarding septic coagulopathy [66]. In 2020, Wang et al. published a study that showed that microRNA material is related to sepsis prognosis [67]. In their study, the authors sequenced plasma microRNA in ‘sepsis-alone’ patients and in patients with SIC, discovering four microRNAs that were differentially expressed in both subpopulations. Especially the plasma Has-miR-92a-3p concentration was significantly lower in SIC patients and was associated with aPTT, prothrombin activity, fibrinogen concentration, and plasma lipocalin-2 concentration, known also as neutrophil gelatinase-associated lipocalin (NGAL). For each unit of increase in plasma Has-miR-92a-3p, the risk of SIC decreased by 32%, and the joined model for Has-miR-92a micro RNA and NGAL for SIC prediction reached an AUROC of 0.687 (95%CI 0.578–0.782, *p* = 0.003). Therefore, they concluded that the Has-miR-92a and NGAL concentration may be used to distinguish SIC patients from sepsis-alone patients and predict the risk of SIC in sepsis patients [67].

### 4.4. Inhibitor of Plasminogen Activator Inhibitor-1 (PAI-1)

Embelin is a natural product that inhibits PAI-1, which possesses both anti-inflammatory and antithrombotic properties and may be useful in sepsis-associated DIC. In a septic mice model, embelin ameliorated inflammation and reduced the formation of microthrombi in the lungs [68].

## 5. Authors’ Perspective on Practical Management Approaches in Different Clinical Scenarios

### 5.1. Patients with SIC

As disseminated microthrombi formation is the main feature of septic coagulopathy, anticoagulation is the treatment of choice here. The most commonly used anticoagulants are UFH and LMWH. Unfractionated heparin is a time-honored anticoagulant, the effect of which can be easily monitored with aPTT. Moreover, there is a specific antidotum for UFH (e.g., protamine sulfate). The disadvantage of UFH use is a risk of heparin-induced thrombocytopenia and thrombosis syndrome (HITTS), which aggravates thrombocytopenia already present in these patients. Moreover, HITTS requires the use of alternative anticoagulants (e.g., fondaparinux, argatroban, and bivalirudin), against which no antidota are available. The lack of antidota may have particular importance in the setting of dynamic coagulation status changes, which occur during septic coagulopathy. The anticoagulant effect of LMWH is more predicted than for UFH and usually does not require laboratory monitoring. The risk of HITTS with LMWH is much lower than for UFH. The most recent SSC guidelines recommend prophylactic anticoagulation with LMWH [6]. At this stage of septic coagulopathy, the authors would preferentially use LMWH in high prophylactic doses (e.g., enoxaparin 30 mg SC twice a day in patients with creatinine clearance > 30 mL/min.). In our opinion, the adjustment of the LMWH dose may depend on the viscoelastic tests of coagulation (ROTEM and TEG).

### 5.2. Patients with Early Sepsis-Associated DIC

According to the Japanese Clinical Practice Guidelines for Management of Sepsis and Septic Shock 2020, at this stage of septic coagulopathy, the use of AT or rhTM is suggested. There is also a suggestion not to use heparin or heparin analogs [7]. Taking into account the cost, in our opinion, these medications should be reserved for sepsis patients with overt DIC (ISTH criteria). Before starting a patient on AT, its serum activity should be measured, and supplementation should be aimed at AT activity ≥ 70 [45].

### 5.3. Patients with Late Sepsis-Associated DIC

In patients with late sepsis-associated DIC, the consumption of PLT and coagulation factors may lead to bleeding. If bleeding occurs, management is shifted toward the replenishment of PLTs and coagulation factors, and the use of antifibrinolytic agents (e.g., tranexamic acid) in patients with hyperfibrinolysis. The point-of-care viscoelastic tests of coagulation (ROTEM and TEG) have an important role here. The use of viscoelastic tests allows for the early differential diagnosis of coagulopathy, which leads to more timely control of bleeding and decreased use of blood products. It is especially important in patients with septic coagulopathy, as the transfusion of blood components increases the risk of thrombosis. The transfusion of blood components may also be required before invasive procedures/surgery in these patients. In this context, the suggested therapy is to aim at PLT > 50 000/µL (platelet concentrate), INR < 2 (fresh-frozen plasma), aPTT < 2 times the upper limit (fresh-frozen plasma), fibrinogen > 150 mg/dL (cryoprecipitate and fibrinogen concentrate) [7].

### 5.4. Patients with Purpura Fulminans

Purpura fulminans (PF) is a rare life-threatening syndrome in which severe DIC results in cutaneous purpura; however, other locations may be affected as well, leading to the amputation of extremities and significant scarring in affected locations. In meningococcal disease, PF was shown to increase mortality from 15% to 20–60% [69]. Immunologically compromised patients (e.g., asplenic or immunosuppressed patients) are at particular risk [70]. The main pathophysiological role in PF is attributed to the severe deficiency of protein C (PC) and the dysfunction of the PC–thrombomodulin pathway. Current treatment is focused on therapeutic anticoagulation and the repletion of natural anticoagulants [71].

## 6. Conclusions

The management of septic coagulopathy is currently an area of intense research. Anticoagulants seem to be beneficial in SIC and sepsis-associated DIC. The most researched anticoagulants in this clinical setting are heparins, antithrombin, and recombinant human thrombomodulin. The last two have been associated with the highest survival improvement. The indiscriminate use of anticoagulants in sepsis patients should be avoided due to the lack of mortality benefit and increased risk of bleeding. The early diagnosis of SIC and monitoring of coagulation status during sepsis is crucial for the timely management and selection of the most suitable treatment at a time. New directions in septic coagulopathy include new diagnostic biomarkers, dynamic diagnostic models, genetic markers for SIC management, and new therapeutic agents. These new research avenues may potentially result in timelier SIC diagnosis and improved management of all stages of septic coagulopathy by making it more effective, safe, and personalized.

## Figures and Tables

**Table 1:** International Society on Thrombosis and Hemostasis sepsis-induced coagulopathy diagnostic scoring system. Based on [2].

Parameter	0 Points	1 Point	2 Points
PLTs ^1^ (×10^9^/L)	≥150	100–149	<100
INR ^2^	≤1.2	1.3–1.4	>1.4
SOFA ^3^ score (points)	-	1	≥2

^1^ Platelet count; ^2^ international normalized ratio; ^3^ Sepsis Organ Failure Assessment—summation of respiratory, cardiovascular, hepatic, and renal components.

**Table 2:** International Society on Thrombosis and Hemostasis disseminated intravascular coagulation diagnostic scoring system. Based on [3].

Parameter	0 Points	1 Point	2 Points	3 Points
Fibrin-related marker	No increase	-	Moderate increase	Severe increase
PLTs ^1^ (×10^9^/L)	≥100	50–100	<50	-
Prolonged PT ^2^ (s)	<3	3–5	≥6	-
Fibrinogen (g/L)	≥1	<1	-	-

^1^ Platelet counts; ^2^ prothrombin time.

**Table 3:** Japanese Association for Acute Medicine disseminated intravascular coagulation diagnostic scoring system. Based on [5].

Parameter	0 Points	1 Point	2 Points	3 Points
FDPs ^1^ (µg/mL)	≤10	10–25	-	≥25
PLTs ^2^ (×10^9^/L)	≥120	80–120 or >30%↓/24 h	-	<80 or >50%↓/24 h
Fibrinogen (g/L)	>1.5	1.0–1.5	<1.0	-
INR ^3^	<1.2	≥1.2	-	-
SIRS ^4^ criteria (number)	0–2	≥3		

^1^ Fibrin degradation products; ^2^ PLTs—platelet count; ^3^ international normalized ratio; ^4^ systemic inflammatory response syndrome.

## Data Availability

Not applicable.
